# Harmonic radiation contribution and X-ray transmission at the Small Quantum Systems instrument of European XFEL

**DOI:** 10.1107/S1600577523003090

**Published:** 2023-05-10

**Authors:** Thomas M. Baumann, Rebecca Boll, Alberto De Fanis, Patrik Grychtol, Markus Ilchen, Ulf Fini Jastrow, Masahiro Kato, Christoph Lechner, Theophilos Maltezopoulos, Tommaso Mazza, Jacobo Montaño, Valerija Music, Yevheniy Ovcharenko, Nils Rennhack, Daniel E. Rivas, Norio Saito, Philipp Schmidt, Svitozar Serkez, Andrey Sorokin, Sergey Usenko, Jiawei Yan, Gianluca Geloni, Takahiro Tanaka, Kai Tiedtke, Michael Meyer

**Affiliations:** a European XFEL, Holzkoppel 4, 22869 Schenefeld, Germany; b Deutsches Elektronen-Synchrotron DESY, Notkestr. 85, 22607 Hamburg, Germany; c National Institute of Advanced Industrial Science and Technology (AIST), NMIJ, Tsukuba 305-8568, Japan; d Institut für Physik und CINSaT, Universität Kassel, Heinrich-Plett-Strasse 40, 34132 Kassel, Germany; Uppsala University, Sweden

**Keywords:** free-electron lasers, X-ray beam transport, harmonic radiation, atomic, molecular and optical science

## Abstract

Transmission measurements of the soft X-ray beamline to the Small Quantum Systems (SQS) scientific instrument at the SASE3 undulator of European XFEL are presented. Measurements are reported for a wide range of photon energies (650 eV to 2400 eV), using X-ray gas monitors as well as a bolometric radiometer. The results are in good agreement with simulations for the beam transport and show a transmission of up to 80% over the whole photon energy range. The contribution of second- and third-harmonic radiation of the soft X-ray undulator is determined at selected photon energies by performing transmission measurements using a gas absorber to provide variable attenuation of the incoming photon flux. A comparison of the results with semi-analytic calculations for the generation of free-electron laser pulses in the SASE3 undulator reveals an influence of apertures along the beam transport on the exact harmonic content to be accounted for at the experiment. The second-harmonic content is measured to be in the range of 0.1% to 0.3%, while the third-harmonic contributed a few percent to the SASE3 emission. For experiments at the SQS instrument, these numbers can be reduced through specific selections of the mirror reflection angles.

## Abstract 2

Measurements of the transmission as well as the contribution of harmonic radiation at the soft X-ray beamline of the Small Quantum Systems instrument at the SASE3 undulator of European XFEL are presented.

## 1. Introduction

The Small Quantum Systems (SQS) instrument is located behind the soft X-ray undulator SASE3 of the free-electron laser European XFEL (Decking *et al.*, 2020 ▸). It is dedicated to studying the interaction of X-rays with atomic and molecular systems as well as nanoparticles and clusters, focusing particularly on non-linear processes, time-resolved femtosecond dynamics and coherent imaging (Mazza *et al.*, 2020 ▸; Eichmann *et al.*, 2020 ▸; Jahnke *et al.*, 2021 ▸; Boll *et al.*, 2022 ▸; Feinberg *et al.*, 2022 ▸; Grychtol *et al.*, 2021 ▸; Rivas *et al.*, 2022 ▸). Such studies require an extreme X-ray photon density, which can uniquely be delivered by free-electron lasers (FELs). Consequently, a good knowledge of the fluence, defined as the number of photons per unit area, is required for planning and performing such experiments.

The fluence is determined by the total energy in each FEL pulse and by the ability to focus these pulses onto the sample. In this article, we concentrate on the FEL pulse energy and investigate the transmission of the X-ray optics delivering the FEL beam to the experimental station at SQS. The energy of the X-ray pulses is measured by X-ray gas monitor detectors (XGMDs), a technique based on photoionization of a dilute gas that was established at FLASH (Tiedtke *et al.*, 2008 ▸). XGMDs are now routinely used at X-ray FELs like LCLS (Tiedtke *et al.*, 2014 ▸), SwissFEL (Juranić *et al.*, 2018 ▸) and European XFEL (Grünert *et al.*, 2019 ▸). Distributing several of these devices along a beamline is a tool to measure the transmission of the X-ray optics, as previously done, for example, at FLASH (Wellhöfer *et al.*, 2007 ▸) or LCLS (Moeller *et al.*, 2015 ▸). An alternative approach for an absolute determination of the FEL intensity is bolometric techniques that measure temperature changes induced by the X-ray radiation in an absorber (Kato *et al.*, 2010 ▸; Tanaka *et al.*, 2015 ▸, 2017 ▸). In this article we utilize both methods.

The fluence at the experiment is dominated by the radiation at the fundamental wavelength of the FEL but also contains contributions from harmonics. For planar undulators as used at SASE3, the most prominent contribution is expected for the third harmonic (Saldin *et al.*, 2006 ▸). However, also the second harmonic can occur at non-negligible levels as calculated by Geloni *et al.* (2007 ▸) or measured at FLASH (Düsterer *et al.*, 2006 ▸) and LCLS (Ratner *et al.*, 2011 ▸). The harmonic radiation can be problematic for certain types of experiments, *e.g.* measurements of non-linear multi-photon processes induced by the fundamental radiation competing with an inseparable one-photon channel of the harmonic (LaForge *et al.*, 2021 ▸). Therefore, we deploy the techniques used for measuring the beamline transmission to investigate the harmonic contribution at the SQS instrument in the second part of this article.

## 2. SASE3 and SQS beamline layout

**Figure 1:**
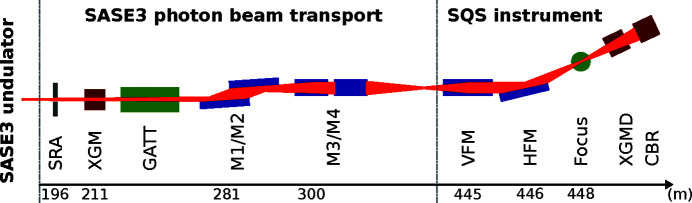
Schematic view of the X-ray beam transport from the undulator exit to the SQS instrument, seen from the top. The mirrors M1, M2, M3, M4, as well as the SQS KB (VFM and HFM), are depicted in blue. The slit system (SRA), the gas attenuator (GATT) and the intensity diagnostics (XGM) are shown as well. Downstream of the SQS interaction zone (Focus) an additional intensity monitor (XGMD) and a compact room-temperature bolometric radiometer (CBR) were installed for the measurements presented here. Positions are given as distance from the end of the undulator. The figure is not to scale.

SQS is one of three scientific instruments at the SASE3 soft X-ray undulator branch (Tschentscher *et al.*, 2017 ▸). The photon beam transport section between the undulator and the SQS instrument is about 450 m long and is schematically shown in Fig. 1 ▸. It contains four grazing-incidence X-ray mirrors that are relevant for the photon beam delivery to SQS. Mirrors M1 and M2 introduce a horizontal chicane to prevent unwanted high-energy radiation propagating to the instrument. Their incidence angle can be tuned between 9 mrad and 20 mrad to change their geometric acceptance and high-energy cut-off. Furthermore, the surface of M2 is bendable, which allows for focusing the beam to an intermediate horizontal focus along the beam transport path. A second pair of mirrors (M3 and M4) is part of the soft X-ray monochromator (Gerasimova *et al.*, 2022 ▸). The cylindrical pre-mirror M3 deflects the beam vertically and focuses it onto the exit slit at a distance of 400 m from the last undulator segment. Either a diffraction grating for monochromator operation or a flat mirror can be inserted in position M4. M3 can also be retracted completely to let the FEL pass to SQS without using M3 and M4. For all measurements described in this article, we utilize a beam transport configuration with M1 and M2 only. This configuration is intended to provide the highest number of photons for example for studies of non-linear phenomena since transmission is maximized by using a minimum number of optical elements.

At the SQS instrument, the photon beam is micro-focused by a pair of mirrors in Kirkpatrick–Baez (KB) configuration named vertical focusing mirror (VFM) and horizontal focusing mirror (HFM). After using a pair of interim mirrors with a fixed elliptical curvature during the first year of operation, a new set of bendable mirrors was installed in 2020. These bendable KB mirrors allow for moving the focus point between two experiment interaction zones at 1.8 m and 3.8 m distance from the second mirror by changing the curvature of the elliptical mirror surfaces. The measurements presented here were made using the bendable mirrors corresponding to the final configuration of the SQS beamline layout. A detailed description of the SASE3 and SQS photon beam transport can be found in the literature (Sinn *et al.*, 2012 ▸; La Civita *et al.*, 2014 ▸; Tschentscher *et al.*, 2017 ▸). Mazza *et al.* (2023 ▸) describe and characterize the beam transport system including the fixed-curvature mirror KB set-up.

## 3. Beamline transmission measurement

The transmission of the photon beam from the undulator to the interaction region at SQS is determined by two main factors: the reflectivity of the mirrors and the geometric aperture of the elements in the beam transport. M1, M2, VFM and HFM are made from large silicon substrates with an 800 mm-long reflective surface that was polished to a tangential height error of about 2 nm peak-to-valley and coated with 50 nm of boron carbide (B_4_C) (Vannoni & Freijo-Martin, 2019 ▸). Depending on the incidence angle and photon energy, each mirror reaches a calculated reflectivity of above 90%. The geometric aperture of the mirrors becomes a relevant factor for photon energies below 1500 eV due to the increasing divergence of the FEL beam with decreasing photon energy, causing the FEL to overfill the reflective surface. This will not only reduce transmission but can also affect the wavefront purity and reduce the quality of the focused beam. One strategy to mitigate this factor is to increase the incidence angle to a maximum of 20 mrad for lower photon energies. Further parameters that can influence the transmission are the vacuum level throughout the beam transport as well as contaminations on mirror surfaces. The entire beam transport section is kept under ultra-high-vacuum conditions with pressures between 1 × 10^−9^ mbar and 1 × 10^−7^ mbar, which minimizes photoabsorption by residual gas. Special care is taken for vacuum chambers housing mirrors, which are treated under particle-free conditions to prevent contamination from dust particles. The possible influence of contamination is discussed – to some extent – below.

We determined the transmission of the beam transport by comparing the FEL pulse energy coming from the undulators with the pulse energy measured downstream of the interaction region at SQS. An X-ray gas monitor (XGM) is installed upstream of the SASE3 beam transport section to measure the incoming FEL pulse energy. The XGM is a larger version of the XGMD and was developed for hard X-ray FELs like European XFEL (Maltezopoulos *et al.*, 2019 ▸). The pulse energy measurement is based on photoionization of a noble gas. The XGM records a current of photo-ions under well known conditions and thereby allows for an absolute determination of the incoming FEL pulse energy using semi-empirical ionization cross sections and photo-ion charge state distributions. Furthermore, it can provide a pulse-resolved signal by recording the emitted photo-electrons, which is calibrated to the ion-based pulse energy measurement (Sorokin *et al.*, 2019 ▸). The data described here solely rely on the ion measurement. A mobile version of the XGMD was installed downstream of the SQS interaction zone to record the FEL pulse energy after beam transport. Both gas monitors were operated with xenon as target gas at a pressure in the mid-10^−6^ mbar range.

For a second, independent measurement a compact room-temperature bolometric radiometer (CBR) was mounted downstream of the mobile XGMD, behind the SQS instrument. This device provides an absolute measurement of the average FEL power by measuring the temperature change in a gold absorber resulting from photon energy deposition (Tanaka *et al.*, 2015 ▸, 2017 ▸). Using the known number of FEL pulses per second, the average pulse energy can be derived from this measurement. The radiometer has an aperture of 5 mm diameter, which puts a constraint on the measurement since under normal operation conditions the beam is larger at the position of the radiometer. Therefore, we closed a slit system upstream of the beamline (including the XGM, *cf*. Fig. 1 ▸), called the synchrotron radiation aperture (SRA), to an aperture of 1 mm × 1 mm to prevent an overfilling of the CBR aperture. This measure minimized the contribution of limited geometric apertures of the beamline elements to the transmission since only the center areas of the mirrors were illuminated. It reduced the incoming pulse energy of a few millijoules by about a factor of three.

**Figure 2:**
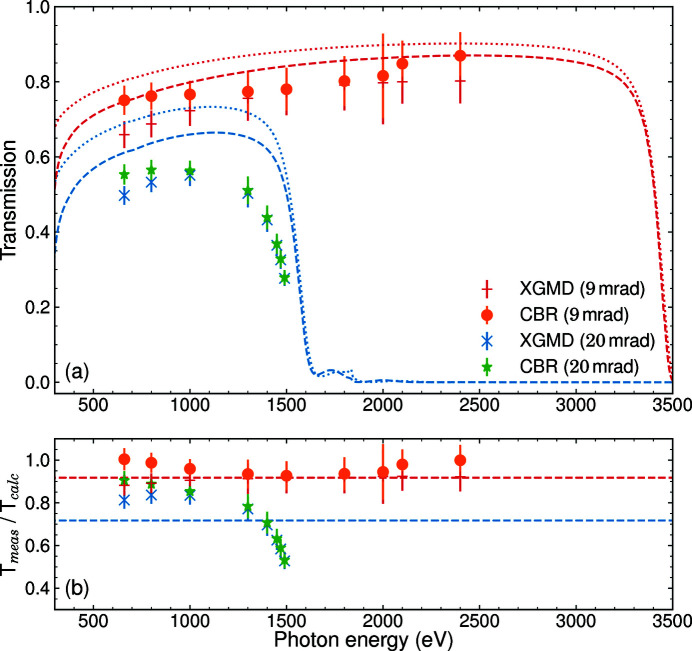
Results of the transmission measurements. (*a*) The data taken by the CBR and the XGMD relative to the upstream XGM, with the FEL confined by the slit system to 1 mm × 1 mm. Data were taken at two beamline configurations with M1 and M2 at 9 mrad and 20 mrad, respectively. The calculated reflectivity of all involved mirrors combined using a 50 nm-thick B_4_C layer is represented by the dotted lines, while the dashed lines include a 5 nm-thick layer of carbon contamination. (*b*) The ratio of the data points and the calculation result for 20 mrad and 9 mrad, including contamination. The dashed lines represent the average ratio beased on the XGMD data.

Fig. 2 ▸(*a*) shows the resulting transmission for the incidence angle of the mirrors M1 and M2 set to 9 mrad as well as 20 mrad, while the M2 surface was kept flat (no intermediate horizontal focus). VFM and HFM were always operated under an incidence angle of 9 mrad. Each point represents the FEL pulse energy detected by the CBR or the XGMD, normalized to the incoming pulse energy measured in the upstream XGM, and averaged over several minutes. The FEL was operated with one pulse per pulse train and a train repetition rate of 10 Hz. The error of the transmission is dominated by the systematic uncertainty of the XGM and XGMD measurements, which lies between 5% and 10% depending on the photon energy (Sorokin *et al.*, 2019 ▸). The uncertainty of the CBR measurement is below 1% (Tanaka *et al.*, 2017 ▸).

A transmission between 70% and 80% was measured in the photon energy range between 650 eV and 2400 eV at the 9 mrad set point. The limits of this photon energy range were given by the *K* parameter range of the variable-gap undulators at an electron beam energy of 14 GeV. At 20 mrad, the transmission at low photon energies was reduced to about 50% and a clear cut-off can be seen at 1400 eV. This also leads to the suppression of the higher harmonics at these higher incidence angles. The results from the XGMD and CBR are generally in good agreement, but show a discrepancy at lower energies due to yet unknown reasons. We calculate the reflectivity of the single-layer mirrors using the CXRO database (Henke *et al.*, 1993 ▸). The dotted lines in Fig. 2 ▸(*a*) represent the calculated transmission obtained from the nominal reflectivity of the mirrors for linear polarized light, using a single B_4_C layer of 50 nm thickness coated on the silicon mirror substrates with a surface roughness of 0.3 nm. The calculations account for a coating density of 2.37 g cm^−3^, as has been previously measured for these mirrors by Störmer *et al.* (2018 ▸). The transmission measurements for the 9 mrad incidence angle of M1 and M2 were taken in a monotonous regime resulting in about 90% of the transmission expected by the calculation. The FEL beam cut-down by the SRA only illuminates the center part of the mirrors, which has been irradiated the most during the past two years of FEL operation. The mirror coating might therefore already be slightly deteriorated in this center area by carbon contamination. To estimate the effect of such contamination, we utilize the two-layer calculation of CXRO and add a 5 nm-thick layer of carbon on top of the B_4_C for all mirrors. The resulting transmission is plotted as dashed lines in Fig. 2 ▸(*a*). Fig. 2 ▸(*b*) further shows the ratio between the data points and the contamination calculation. The choice of 5 nm thickness is based on measurements of the carbon contamination layer thickness on top of the previously used KB mirrors with fixed curvatures after they were taken off the beamline. The inclusion of a carbon layer improves the agreement between calculation and measurement. However, there is still a deviation especially for the data taken at the incidence angle of 20 mrad. Here we are also probing the transmission cut-off, a region where the calculation is very sensitive to its input parameters. The average ratio between measurement and calculation is thereby dominated by the deviation in the cut-off region. The actual B_4_C layer thickness and density, but also the actual incidence angles of all mirrors, can differ from the numbers we use for the reflectivity calculation. This might be the reason for the remaining deviation.

**Figure 3:**
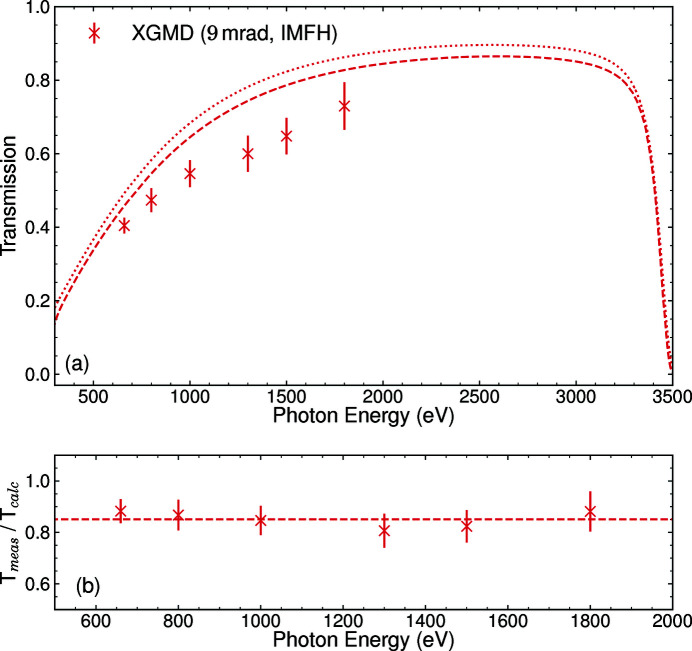
Results of the transmission measurement with an open SRA. (*a*) The data taken by the XGMD relative to the upstream XGM using a beamline configuration with M1 and M2 at 9 mrad and an intermediate horizontal focus. The dashed line represents a calculated transmission combining mirror reflectivity including carbon contamination and geometric aperture. The dotted line uses the nominal reflectivity without contamination. (*b*) The ratio of the data points and the calculation result with their average as a dashed line.

Opening the SRA to 4.5 mm × 4.5 mm lets the full FEL beam enter the transport section and allows for measuring the beamline transmission including the contribution from its geometric acceptance. The results using only the XGMD are shown in Fig. 3 ▸. For this measurement, M1 and M2 were set to an incidence angle of 9 mrad and the bending mechanism of M2 was used to create an intermediate horizontal focus (IMFH) at a position of about 400 m along the beamline. The IMFH decreased the horizontal size of the beam to a few millimetres directly upstream of the KB system, making it smaller than the clear aperture of the HFM. This leads to a reduction of losses due to the limited aperture of the KB system. A calculation of the transmission for this scenario uses the product of mirror reflectivity and geometric mirror acceptance. The reflectivity is derived without (dotted line) and including (dashed line) carbon contamination, as discussed above. For calculating the geometric acceptance, a model of the FEL beam divergence derived by Schneidmiller & Yurkov (2011 ▸) is used. The result is shown as the dashed line in Fig. 3 ▸(*a*). When compared with Fig. 2 ▸, it becomes evident that using the full beam provides a lower transmission for energies below 1500 eV. This is due to the geometric aperture of the mirrors becoming smaller than the beam (which has a larger divergence at lower photon energies). This effect is included in the calculated transmission. However, as seen in Fig. 3 ▸(*b*), the measured transmission yields about 85% of the calculated one, which is a deviation larger than seen in the reflectivity measurements. This is possibly a result of the uncertainty in the calculation for the FEL divergence, which serves as a basis for the calculated geometric acceptance. Another source of uncertainty is the actual position of the IMFH, which might deviate from the one used in the calculation. Taking the deviation into account one can still use the reflectivity calculations as reference for the overall transmission of the beamline.

## 4. Harmonics contribution measurement

The contribution of the harmonics to the FEL radiation can be determined with the bolometric radiometer in combination with the gas attenuator in the SASE3 beamline, making use of the different photo-absorption cross sections for the harmonic and fundamental. The gas attenuator is a 15 m-long cell upstream of the beam transport mirrors (see Fig. 1 ▸), which is filled with a gas (in this case N_2_) to a pressure of up to 10 mbar to attenuate the FEL beam (Sinn *et al.*, 2012 ▸).

The transmitted intensity *I* through the gas attenuator can be described by 

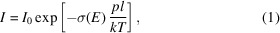

with incoming intensity *I*_0_, photon energy *E* dependent absorption cross section σ(*E*), gas pressure *p*, gas temperature *T* and length *l* of the gas attenuator as well as Boltzmann’s constant *k*. We model the FEL fundamental and harmonics as a superposition of *n* discrete spectral components with a relative contribution *P*_*i*_ to the incoming intensity *I*_0_. For calculating the transmission through the entire beamline, the energy-dependent mirror transmission *M*_*i*_ has to be included. Thus, the transmission in dependence of the attenuator pressure is

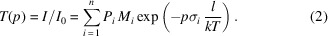

From this we can determine the contribution of the harmonics *P*_*i*_ by measuring the transmission using the XGM and the CBR at different pressures in the gas attenuator. In the following we will measure the harmonic contribution, which scales with the FEL fundamental energy, at a few selected points.

**Figure 4:**
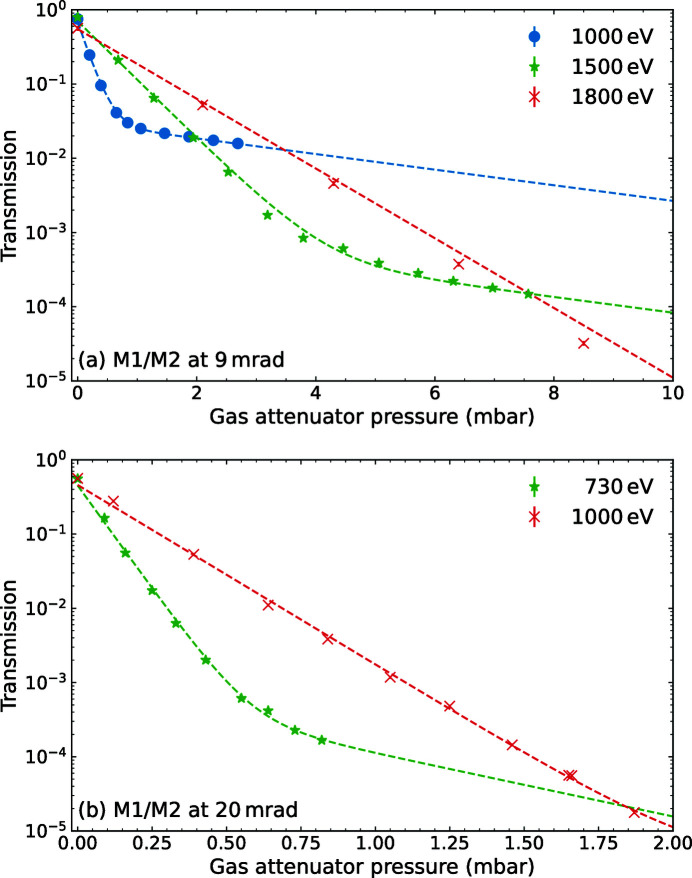
Results of the harmonics measurements. (*a*) The transmission measured with the CBR relative to the upstream XGM as a function of the gas attenuator pressure for M1 and M2 at an incidence angle of 9 mrad. The dashed lines represent the results of a two-parameter fit. (*b*) Data for an M1/M2 incidence angle of 20 mrad for 730 eV and 1000 eV.

For investigating the second-harmonic contribution, the mirrors M1 and M2 were set to an incidence angle of 9 mrad, which suppresses photons of energies above 3500 eV [see red dashed line in Fig. 2 ▸(*a*)]. The undulators were producing FEL radiation at a fundamental energy of 1500 eV. The transmission was determined as the average ratio of the incoming FEL pulse energy and that measured in the CBR in dependence of the pressure in the gas attenuator. During these measurements, the FEL was operated with a gradually increasing number of pulses per pulse train, starting from one at low gas attenuator pressure up to 400 at high pressure. This was done to keep the measured average power and thereby the acquisition time roughly constant. The green stars in Fig. 4 ▸(*a*) show the results. Equation (2) describes the shape of the curve: at *p* = 0 the transmission is defined by the mirrors and corresponds to the results of the transmission measurement in Fig. 2 ▸. At low gas pressures, the slope is dominated by the absorption cross section of the fundamental. At high pressures, the fundamental radiation is mostly absorbed in the gas attenuator and thereby the cross section of the harmonic radiation determines the slope. To quantify the contributions under the assumption of two components, we derive a fit function from equation (2),

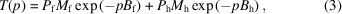

with the mirror transmission *M*_*i*_ taken from the transmission measurements. The attenuation coefficients *B*_*i*_ = σ_*i*_ *l*/(*kT*) are determined from the known length *l* and temperature *T* of the gas attenuator using the photo-absorption cross section σ_*i*_ for N_2_ calculated with scattering coefficients from the literature (Henke *et al.*, 1993 ▸) as shown in Table 1 ▸. This leaves two free fit parameters *P*_f_ and *P*_h_ that represent the contributions of the fundamental and harmonic. The dashed green line in Fig. 4 ▸(*a*) shows the result of the fit from which we obtain a contribution of *P*_2_/*P*_1_ = 0.14 ± 0.02% for the second harmonic. The third harmonic is absorbed by the mirrors.

To measure the third-harmonic contribution, the photon energy of the FEL was decreased to 1000 eV. The resulting pressure scan is shown as blue dots in Fig. 4 ▸(*a*). Applying the two-parameter fit containing the fundamental and third harmonic, we obtain a harmonic contribution of *P*_3_/*P*_1_ = 4.4 ± 0.4%. The second harmonic is also present in this 1000 eV measurement, but is assumed to be at least an order of magnitude weaker than the third harmonic – as seen at 1500 eV. The fit method cannot extract the second harmonic contribution from this data by adding an additional term to equation (3); it might only contribute to the uncertainty of the result. An alternative approach to fit the 1000 eV data would be the use of a four-parameter fit with the additional free fit parameters *B*_f_ and *B*_h_ from equation (3). Such a fit yields a very similar result of *P*_3_/*P*_1_ = 4.2 ± 0.4%. However, the four-parameter fit is not converging for any of the other data sets with fewer data points in the high-pressure regime, therefore we use the two-parameter fit instead.

One data set was taken at 1800 eV, where the second harmonic is absorbed by the mirrors as well [red crosses in Fig. 4 ▸(*a*)]. Indeed, the transmission shows the exponential decrease of the fundamental intensity and does not indicate the contribution of a second energy component up to the maximum applicable pressure, yielding *P*_2_/*P*_1_ = 0 in the fit.

Fig. 4 ▸(*b*) shows additional measurements taken at an incidence angle of 20 mrad on M1 and M2. The increased angle leads to an energy cut-off at about 1600 eV which allows for an investigation of the harmonic contribution at lower photon energies. Here, we measure a second harmonic contribution of *P*_2_/*P*_1_ = 0.27 ± 0.04% at 730 eV and see that we can suppress the harmonics at 1000 eV.

The results of the harmonics measurements are summarized in Table 2 ▸. The error bars are statistical and result from the fits. Overall, we find the contribution of the third harmonic to be on the level of a few percent, while the second harmonic is within 0.1% to 0.3% of the fundamental. Compared with measurements made at LCLS (Ratner *et al.*, 2011 ▸) around a FEL photon energy of 1 keV, our harmonic content is higher. In the following, we will further analyze the experimental results by comparison with calculations and show the dependence of the harmonic content on the fundamental photon energy.

## 5. Calculation of harmonics contribution

The harmonic content of the FEL emission with the FEL tuned at the fundamental pertains a radiation mechanism known as non-linear harmonic generation (NHG) (Huang & Kim, 2000 ▸; Freund *et al.*, 2000 ▸; Tremaine *et al.*, 2002 ▸; Biedron *et al.*, 2002 ▸). The FEL is driven by the fundamental, but the bunching develops higher harmonics, which then radiate. NHG on even harmonics follows a different mechanism compared with odd ones, with the angle-integrated power depending on the FEL diffraction parameter. Once the bunching is known, the generation of harmonic radiation is an electrodynamical process which can be calculated analytically from Maxwell’s equations. A detailed derivation of the harmonic properties was carried out by Geloni *et al.* (2007 ▸), where the authors obtain the power *P*_2_ of the second harmonic relative to the fundamental *P*_1_ using 

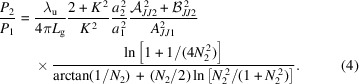

The third-harmonic contribution can be derived in an analogous way to be 

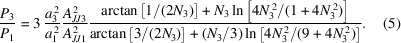

In the previous equations we used the following notation: *K* is the undulator parameter, λ_u_ the undulator period, *L*_g_ the gain length of the fundamental and *N*_*h*_ the Fresnel number for the radiation of harmonic *h*,

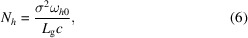

where the transverse size of the electron beam is σ, the speed of light *c*, and the frequency of the harmonic ω_*h*0_. Moreover, for odd harmonics (*h* odd), we define 

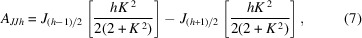

while for even harmonics (*h* even) 

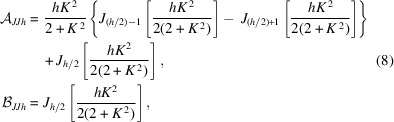

indicating with *J*_*n*_ the Bessel functions of the first kind and order *n*. The microbunching of the electrons in the field of the fundamental radiation is defined through the bunching factors *a*_*h*_, which describe the density modulation level at the fundamental (*h* = 1) as well as at the target harmonic, and have to be known for solving the electrodynamical problem.

The bunching ratios *a*_2_/*a*_1_ and *a*_3_/*a*_1_ for the second and third harmonic can be retrieved from simulations of the FEL process using the experimental conditions as input. To this end, we utilize both the *GENESIS* (Reiche, 1999 ▸) as well as the *SIMPLEX* (Tanaka, 2015 ▸) codes. The bunching is averaged over the duration of the electron beam that is assumed to have a flat-top current profile. From both codes we obtain similar results for the undulator tapering conditions used during the experiment, an electron beam with an energy of 14 GeV and a normalized emittance of 0.5 × 10^−6^ as well as an undulator period of 68 mm. These are applicable for all discussed photon energies, 

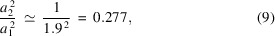




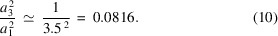

With these bunching ratios we can use equations (4) and (5) to obtain an estimate of the harmonic contribution under the conditions of the experiment. We obtain a second-harmonic contribution of *P*_2_/*P*_1_ = 0.12% at 730 eV and *P*_2_/*P*_1_ = 0.06% at 1500 eV. The third harmonic contribution at 1000 eV is calculated to be *P*_3_/*P*_1_ = 1.9%. The results are summarized in Table 2 ▸ in the ‘Analytical’ columns.

**Figure 5:**
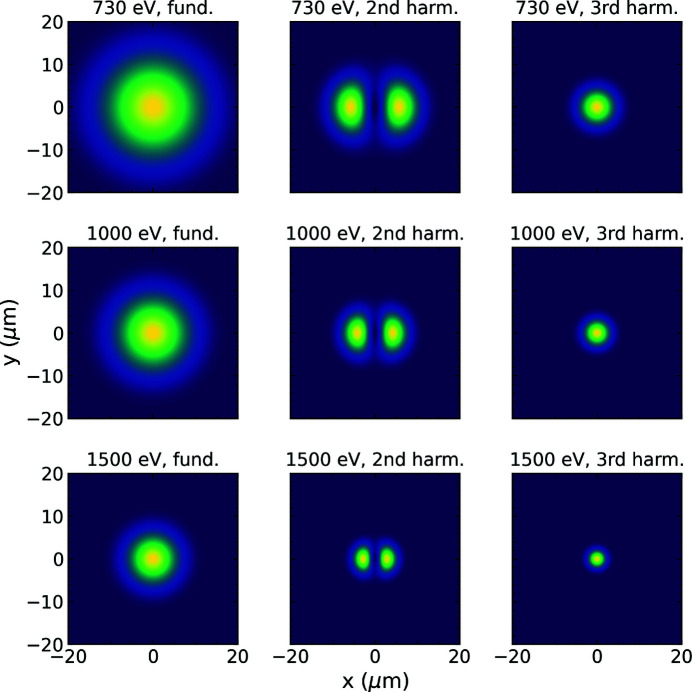
Normalized transverse beam profiles of the fundamental, second- and third-harmonic radiation after the last undulator cell, calculated analytically for three different fundamental energies (730 eV, 1000 eV and 1500 eV).

These calculated harmonic contributions deviate from the measured ones by about a factor of two. As discussed above, the SRA slit system has been closed to a 1 mm × 1 mm aperture to prevent an overfilling of the CBR during the measurements. This has to be taken into account in the calculations. Fig. 5 ▸ shows the normalized transverse beam profiles for the fundamental as well as the harmonic radiation after the last undulator cell for three different fundamental photon energies. The profiles are calculated from the far field radiation obtained in the semi-analytical model. When propagating the divergent beam 196 m further downstream to the SRA, it becomes larger than the aperture. The contributions of fundamental and harmonics are affected differently due to their spatial distributions. The third-harmonic radiation has a smaller divergence than the fundamental and is thereby cut less. Including the aperture in the calculation yields *P*_3_/*P*_1_ = 10.6% at 1000 eV. The second harmonic is emitted off axis, so the aperture leads to an underestimation in the experiment. The calculation results in *P*_2_/*P*_1_ = 0.05% at 730 eV and *P*_2_/*P*_1_ = 0.07% at 1500 eV. These results still deviate from the measurements.

**Figure 6:**
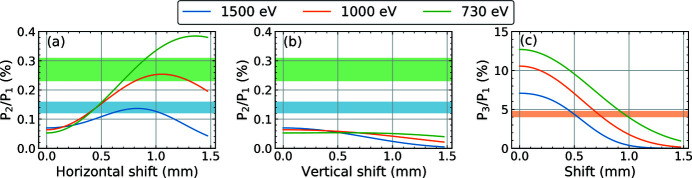
Calculated dependence of the harmonic contribution on a shift of the aperture center from the FEL axis for three different fundamental photon energies (730 eV, 1000 eV and 1500 eV). The aperture has a size of 1 mm × 1 mm and is located 196 m downstream of the undulator. (*a*) Second-harmonic contribution, horizontal shift; (*b*) second-harmonic contribution, vertical shift; (c) third-harmonic contribution, horizontal or vertical shift. The shaded areas represent a 2σ range around the measured values.

The above calculation assumes the SRA to be exactly centered on the FEL axis. This was not the case during the measurements due to slight beam pointing variations between the different photon energy settings. Looking at the calculated beam profiles in Fig. 5 ▸, it is evident that shifting the aperture away from the center of the fundamental leads to a relative change of the harmonic contributions. This is quantified in Fig. 6 ▸, which shows the calculated dependence of the harmonic contributions on a horizontal and vertical center shift of the 1 mm × 1 mm SRA. The shaded areas represent a 2σ range around the values obtained from the measurements. The calculations for the third harmonic suggest an aperture shift of above 0.68 mm from the FEL axis to match the measured values. Such a shift in the horizontal direction also fits the second harmonic data. However, this would correspond to an angular beam shift of about 3 × 10^−3^ mrad and we cannot confirm such a large shift from measurements using beam imagers taken during the beamline preparation. These data show a shift of about 0.4 mm in the horizontal direction and, depending on the beamline configuration, between 0.1 mm and 0.4 mm vertically. In these positions, the calculation deviates from the measured values by about a factor of two.

In summary, the semi-analytical model of the FEL process describes the harmonic content and the effect of a small aperture in the beam transport, reproducing the measurements within a factor of two. It also demonstrates a possibility of controlling the harmonic content with the aperture, at the cost of transmitted pulse energy.

**Figure 7:**
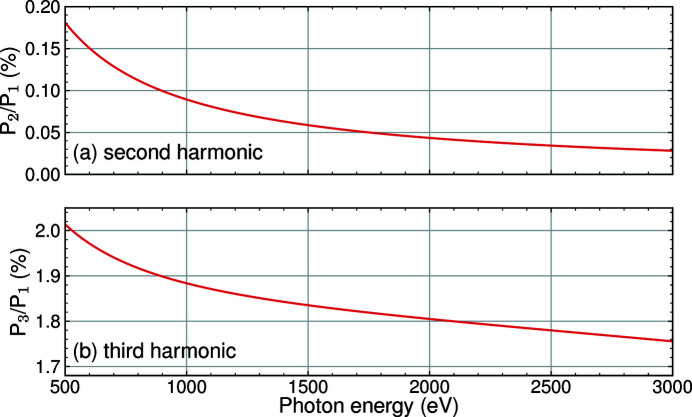
Contribution of the second- (*a*) and third-harmonic (*b*) radiation in dependence of the fundamental photon energy, calculated from the semi-analytical model for the undulator tapering conditions during the harmonics measurements without including apertures.

We can further use the semi-analytical model to calculate the second- and third-harmonic contribution for the entire energy range of the SASE3 undulator. The results are depicted in Fig. 7 ▸. These harmonic contributions are calculated for the undulator tapering conditions used above and do not include any aperture. They demonstrate values expected for typical experimental conditions at SQS, if the harmonics are not cut by the beam transport mirrors.

## 6. Conclusion

The transmission of the X-ray optics in the beam transport section of SASE3 including the M1/M2 horizontal chicane as well as the bendable KB mirrors of the SQS instrument was determined using X-ray gas monitor detectors and a bolometric radiometer. The measurements show a transmission between 50% and 80%, depending on photon energy and transport configuration, which is slightly lower than expected by calculations. The deviation from the calculation might partly be explained by carbon contamination on the mirror surface. Using the same detectors, the contributions of harmonic radiation were determined to be a few percent for the third and 0.1% to 0.3% for the second harmonic. These results can be described by a one-dimensional semi-analytical model of the FEL radiation emission in the undulator.

In general, the exact harmonic contribution depends on the tuning of the trajectory the electron beam takes through the undulators, which can change on a day-to-day basis. Furthermore, apertures along the beam transport have an influence on the harmonic content. For experiments that are sensitive to the harmonic radiation the best option is to suppress their contribution with the X-ray mirrors by choosing a steeper incidence angle. Alternatively, the contribution has to be assessed. The gas attenuator scans can be performed in preparation for an experiment using an XGMD or even a beam imaging unit with a scintillator screen as detector (Ratner *et al.*, 2011 ▸). One option for generating an online signal for optimization and monitoring of the harmonic contribution would be photo-electron spectroscopy: electrons ionized from a rare gas atom by the harmonic radiation can be detected in a time-of-flight spectrometer. The signal from these electrons could then be used to minimize the harmonic contribution. Such spectrometers are available at the SASE3 beamline (Laksman *et al.*, 2019 ▸) as well as SQS (De Fanis *et al.*, 2022 ▸).

## Figures and Tables

**Table 1:** Photo-absorption cross sections of nitrogen for the fundamental and harmonic energies used in the two-parameter fits, calculated from Henke *et al.* (1993 ▸)

*E*_f_ (eV)	σ_f_ (cm^2^)	*E*_h_ (eV)	σ_h_ (cm^2^)
1000	1.54 × 10^−23^	3000	6.68 × 10^−25^
1500	5.02 × 10^−24^	3000	6.68 × 10^−25^
1800	2.99 × 10^−24^	3600	3.86 × 10^−25^
730	3.54 × 10^−23^	1460	5.42 × 10^−24^
1000	1.54 × 10^−23^	2000	2.20 × 10^−24^

**Table 2:** Summary of the measured harmonic contributions at different photon energies

	Experiment		Analytical	
	*P*_2_/*P*_1_	*P*_3_/*P*_1_	*P*_2_/*P*_1_	*P*_3_/*P*_1_
730 eV	0.27 ± 0.04%		0.12%	1.96%
1500 eV	0.14 ± 0.02%		0.06%	1.85%
1000 eV		4.4 ± 0.4%	0.09%	1.91%
